# Wars, Conflicts & Mental Health

**DOI:** 10.12669/pjms.40.5.9505

**Published:** 2024

**Authors:** Afzal Javed

**Affiliations:** 1 Afzal Javed, M.B.B.S; M.C.P.S; D.Psych. (Lond.); Board Cert.Psych. (U.K); M.Phil. (Edin.); F.R.C.Psych. (UK) F.R.C.P (Edinburgh & Ireland). Immediate Past President World Psychiatric Association, Consultant Psychiatrist, Honorary Professor Institute of Applied Health Research, University of Birmingham, UK. Honorary Associate Clinical Professor, Warwick Medical School, University of Warwick, UK. Chairman Pakistan Psychiatric Research Centre, Fountain House, Lahore, Pakistan

The world has never been without wars, violent conflicts and disasters.^1^-^3^ The number of current wars or conflicts in the world today varies depending on the source and the definition of war or conflict. According to Wikipedia, there are around 40 ongoing wars and conflicts in the world. These conflicts lead to several psychological, social, physical, and economic effects that come with severe psychosocial & personal adversities. The families suffer from increased stress for coping in addition to financial strains that contributes significantly to their quality of life.

World Bank in their 2022 report stated that the current wars & violent conflicts may lead to a higher number of refugees who may be internally displaced in their own countries or people with the external displacement leaving their homelands.^4^ World Bank’s group on fragility, conflict, and violence (FCV) estimated that millions of individuals were currently displaced from their homes and this figure may even go to further higher numbers by the end of this decade. As per their approximation, the number of extremely poor people living in the world, may reach to the two third of the global population and most of these will be living in countries and areas that are affected by conflicts.^5^ Children & women are equally the worst sufferers of any domestic or international conflict, fighting or wars. They, unfortunately, do not understand, they do not know what can be done, and they often have no escape. Many wars are taking place in areas that are already struggling for survival of economies and the conflicts & these wars are increasing such sufferings.

Unfortunately, the victims of these conflicts are usually the poor and already from neglected groups fighting with poverty, security, and law & order situations. Looking at the refugee’s status, it is observed that the developing countries are affected more with internal displacements and millions of citizens, including skilled people, claim asylum and shelter in the developed world. Additionally, the conflicts affect health workers and health institutions that may already be suffering from limited manpower, resources, and staffing. Human right issues are likewise neglected & ignored in these areas and affected populations may even get further setbacks of sufferings of inequality and social injustice. Wars, of course, play a vital role in disturbing the routine lives and the fabrics of the society leading to further dysfunctions.

WHO’s Constitution entered into force in 1948. This identifies the link between health and peace, and state that “the health of all peoples is fundamental to the attainment of peace and security and is dependent upon the fullest co-operation of individuals and states”.^6^ World Health Assembly in 1981, again, highlighted these principles and emphasised for preserving and promoting “peace as the most significant factor for the attainment of health for all” and expected WHO member states for the implementation of the UN resolutions on peace and promoting peace for controlling the conflicts (resolution WHA34(38).^7^,^8^

During 1980s WHO came up with further reports advocating provision of health services during conflict & post-conflict times. Next few years witnessed new initiatives by WHO for developing several policies and emphasizing the significance of peace and its impact on health. One of the innovative programmes was named as “Bridge for Peace” that asked UN member countries for considering humanitarian basis and developing plans for improving health infrastructure during conflict & post-conflict timings.^9^

The damage to medical and social systems due to wars, conflicts & violence has multiple consequences.^10^,^11^ Impact on mental well-being, is equally crucial for both physical health & general well-being. Likewise, mental disorders are shaped by social determinants and social disadvantages linked to conflicts and trauma. The slogan that “no health without mental health” is relevant as studies and reports constantly show the significant links between physical & mental health. Scientific evidence continuously demonstrates the troublesome impacts of global conflicts and the ever-increasing lists of traumatic events having a long-lasting effect on mental well –wellbeing.^12^ World Health Organisation, various professional associations and several NGOs also emphasize that in situations of armed conflict, general population become susceptible to negative mental health. Similarly, the prevailing & on-going conflict situations may increase the vulnerability of people to develop further distress and lesser their resilience to fight against these adversities.^13^

In terms of extent of mental health problems, an increased burden has been observed for the number of cases suffering from anxiety, depression, and post-traumatic stress disorders. This certainly raises the importance of provisions of treatment & preventive management approaches in the war affected areas. The acknowledgement of mental health issues as public mental health problems would, therefore, call for availability of appropriate preventive interventions to reduce the prevalence of psychiatric symptoms during the time of conflict & violence. The literature is rich in studies showing the role of traumatic events in increasing risk of developing mental illnesses with depressive disorders as one of the highest contributors to health & social burdens. Similarly, anxiety, psychosomatic problems, post traumatic disorders, and behaviours that may affect the ability to function effectively, also add to the growing burden.^14^

In addition to the civilian population undergoing stress of living in conflict areas, soldiers on both sides of the conflict and those viewing the images of war and conflicts through social media get further vulnerabilities leading to mental health problems along with misery, unhappiness, helplessness and despair.^15^ The Western Psychology views trauma as a personal construct. However, the sufferings from trauma are more than a private experience and need to be conceptualised in a broader social and societal context.^16^ The populations under constant threat generally find that their values for coping the stress are undermined and their cultural and traditional standards to deal with collective trauma & suffering are compromised with risks of collapse of social norms. This leaves them in a state of helplessness, despair, and anguish.

Furthermore, hopes for getting help from others get less leading to added mistrust & apprehensions for further breakdown. Setback for communities’ vital function for social cohesion gets extra challenges of exploitation, breach of human rights and many other psycho-social adversities for all groups of the populations.^17^ Along with many other adversities, the primary impact of war on victims is through their experiences of watching the destruction of their identity, individuality, and distinctiveness. War affected individuals may not be able to imagine personal survival in case their independence & collective pride has been hurt. This concept of “cultural bereavement” describes feeling of continuous guilt about abandoning their homeland and unfulfilled obligations to those who have not lived through these crises.^16^

The damage to medical systems due to wars and conflicts has a host of consequence. This damage is incurred through deliberate targeting of or collateral damage to health facilities. Health professionals should, therefore, acknowledge that violent armed conflicts directly affect health systems and obstructs the delivery of equitable healthcare. While there is no health without mental health,^18^,^19^ the impact of wars and conflicts on the mental well-being of the population are also worth noting. Current evidence does show an increased incidence and prevalence of mental disorders & psychological distress in many wars affected areas, thus, advocating a need for acknowledgement of the extent of the damages and prevalence of mental disorders during the wars and violent conflicts that may also contribute significantly to adverse social & economic aspects of living.^20^-^25^

There is a need to adapt a health and peace approach that should advocate for an understanding of the requirements for planning programmes for ensuring a long-term access to healthcare and mental & physical well-being in targeted countries. Mental health professionals should also understand the role of social contexts and needs for psychological resilience, recovery & empowerment in mental well-being especially during periods of trauma and conflicts.

Finally, as the extent of the problem is enormous, there is an urgent need to develop and implement interventions that should address the local burdens. For any intervention to be effective & operative, it is vital that policies & practices should be locally driven with a focus on local issues, needs and requirements. Local leads, of course, can play a major role in delivering the services rather than relying on international experts who may have limited knowledge about local requirements.

It is a pity that a rise in global wars, conflicts & organised violences are increasing the number of morbidities & mortalities significantly. The future approaches thus require a radical reshaping of our polices including funding for more resources, reviewing national disaster management agendas, encouraging research programmes, sharing details of mental & physical health adversities, and capacity building at institutional and community levels. This, of course, can only be achieved through a review of the national & regional strategies and not relying on out-of-date foreign guidelines, poor local infra-structure & unfair distribution of resources.

Finally it is recommended that long term policies and strategies should be planned to deal with the medical & psycho-social impacts of conflicts and trauma. While national health policies are needed to include disaster management, there should be a priority for reshaping the local, regional & global structures with an emphasis on allocation of resources, evaluation of needs and strengthening of infrastructure to meet the requirements.
